# Effective DC acceleration of charged particles in a circular ring and its potential application

**DOI:** 10.1038/s41598-023-40859-2

**Published:** 2023-08-21

**Authors:** Ken Takayama

**Affiliations:** Accelerator Laboratory, High Energy Accelerator Research Organization (KEK), Tsukuba, Ibaraki 305-0801 Japan

**Keywords:** Engineering, Optics and photonics, Physics

## Abstract

A novel scheme is proposed for realizing effective DC acceleration of charged particles in a circular ring. The key is to use an induction acceleration cell with multi-insulating gaps that allow forced short circuit using solid-state switching devices such as SiC-MOSFET or unforced short circuit using diodes. A baseline design of the induction acceleration cell is shown. A coasting beam is accelerated in the circular ring without bunching. As its possible application, a continuous-wave terahertz free-electron laser (FEL) is proposed with numerical parameters, where this effective DC acceleration compensates for energy loss of the electron beam occupying the entire isochronous storage ring due to synchrotron radiation and FEL interaction. As a result, FEL microbunching of a coasting beam can be created, resulting in a high average output power that has not been possible with existing linac-driven FELs or RF storage-ring FELs.

## Introduction

**Figure 1:**
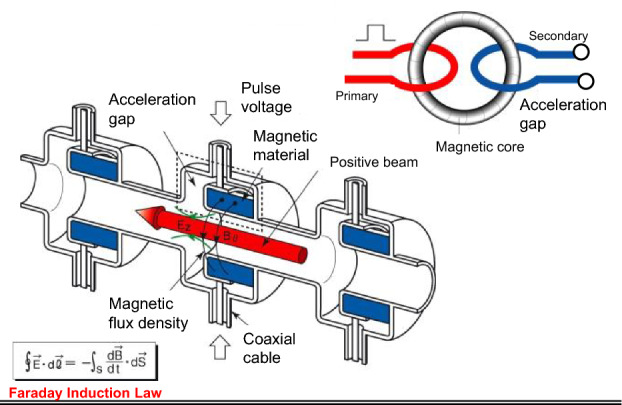
Principle of induction acceleration and analogy from a one-to-one transformer. The primary loop is connected to the switching power supply providing a pulse voltage and the secondary loop corresponds to the beam orbit in a circular accelerator ring. Flux change of the magnetic material in the induction acceleration cell generates an induced voltage across the ceramic gap (acceleration gap).

So far the basic laws of physics have told us that it is impossible to accelerate repeatedly charged particles in DC mode in a circular ring. This paper describes how this long-believed restriction can be effectively overcome. To do this, induction acceleration is used. The induction acceleration device is employed, the working principle of which is governed by Maxwell’s equations not including the term of displacement current. The induction acceleration method has evolved, since it was first proposed by Widerøe in his doctor thesis^1^. Induction acceleration is made possible by an induced voltage across an insulating gap in the induction cell, as shown in Fig. 1. This induced voltage, called a set voltage, is generated synchronously with beam circulation. Here, it must be emphasized that this must always be accompanied by a reset voltage with negative polarity in order to avoid saturation of the magnetic material in the induction cell. During this reset process, the circulating beam is decelerated. Thus, existing induction acceleration systems cannot be used for effective DC acceleration without some modification.

**Figure 2:**
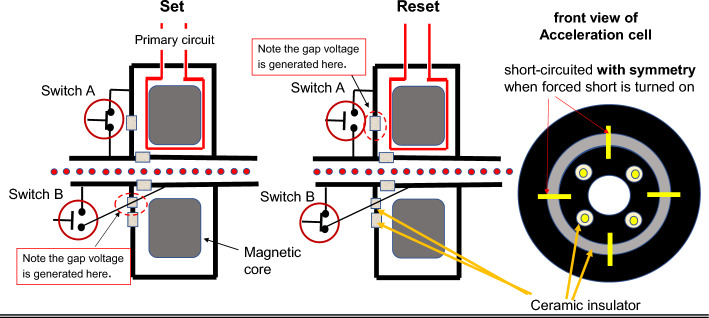
Schematic view of the Induction cell with multi-insulating gaps. Left 2 figures show the schematic side view of the cross-section of the induction cell, where red lines represent the primary current circuit loop surrounding the magnetic core shown with large gray boxes and a row of red dots shows a DC beam or FEL micro-bunches as discussed later. its operational performance in set mode and resent mode. In addition, small gray boxes represent ceramics for insulation. They are welded to metal beam pipes and metal canister of the induction acceleration cell. Right figure depicts its front view and yellows corresponds to a switching metal rod, on the middle of which the active switching device is mounted.

This paper proposes as an alternative an induction cell with multi-shielding gaps as shown in Fig. 2. Programmed excitation of two induction cells of this type applies an effective accelerating DC voltage to charged particles. Consequently, continuous acceleration of a uniform beam stored in a circular ring becomes possible.

RF displacement acceleration of a coasting beam circulating in a circular ring is well known, where a vacant RF bucket is created in the longitudinal phase space accommodating the coasting beam and it is slowly moved into the lower energy region, in association with acceleration of the coasting beam due to Liouville's theorem. This process is repeated until the desired energy is achieved. This acceleration technique was employed in the Intersecting Storage Ring (ISR) at CERN^2^, where a beam injected from the CERN 28 GeV Proton Synchrotron to the ISR was accelerated to 31 GeV. Several undesirable features in RF displacement acceleration are worth noting. Large RF modulation on the coasting beam in the phase space is unavoidable; as a result, the energy spread in the beam rapidly expands when the RF bucket passes through the phase-space area occupied by the beam and an increase in emittance due to non-adiabatic beam handling accompanies this process. In addition, RF displacement acceleration takes time. Thus, the utility of RF displacement acceleration is limited.

The present DC acceleration method can overcome these disadvantageous features and may be an attractive in a wider range of applications. Below, a representative example of possible applications for a continuous-wave (CW) free-electron laser (FEL) in an isochronous storage ring will be introduced, although its feasibility is not fully justified at the present stage.

## Induction cell with multi-insulating gap

The induction cell is a kind of one-to-one transformer, as shown in Fig. 1, and described in many studies^3^. The internal structure of the present induction cell is similar to existing ones, in which a magnetic core (e.g., FINEMET) is encircled by a primary excitation coil and the inner vacuum duct for the beam connected to the cell’s metal cage is equivalent to a secondary loop. The accelerating voltage is induced across the ceramic gap that is part of the vacuum duct. When a voltage pulse is applied across the primary coil’s terminal, the accelerating voltage continues to be induced until the magnetic core is saturated. Core resetting is essential in order to generate the next accelerating voltage pulse. For this purpose, a negative voltage pulse with the same pulse length as that of the set-voltage pulse must be applied across the primary coil’s terminal. The charged beam cannot pass the acceleration gap during the resetting period. This has been a main restriction on the existing induction synchrotrons^4,5^ and other circular induction accelerators^6^.

In the proposed induction cell, another circular ceramic gap is introduced on the front cell-plate so as to shield the electrical conductance of the stainless-steel plate as shown in Fig. 2. Electrical conduction between the two regions of the outer cage of the induction cell are forced into short-circuit or open-circuit configuration by means of multiple metal rods (four are shown in the figure). Further, four relatively small ceramic plates with a brazed joint to other metal rods, both ends of which are connected to the upstream/downstream beam pipe, are located near the beam pipe on the front cell plate. Electrical conduction in all the metal rods is controlled by active switching elements such as SiC-MOSFETs with a high withstand voltage^7^. CST Studio simulations^8^ show that geometrically symmetric short circuits are important for field uniformity within the induction cell.

For the reader’s reference, typical parameters of the 4 kV induction acceleration cell are given in Table 1, which can be easily evaluated from the existing induction cell designs^3^. The design is based on the relation of the time-integrated induced voltage and maximum magnetic flux, $V \cdot T = \Delta B \cdot S$, where *V* is the induced voltage, *T* is the pulse length, Δ*B* is the magnetic flux swing, and *S (d x *Δ*r)* is a cross section of the toroidal-shape magnetic core where *d* and Δ*r* represent the core length and the difference between inner diameter and outer diameter, respectively.

**Table 1:** Parameters of the 4 kV induction cell.

Flux swing width	Δ*B*	2 T
Pulse length	*T*	10 μs
Voltage height per cell	*V*	4 kV
Required core size	*S* (*d* × Δ*r*)	0.2 m × 0.2 m
Repetition rate	*F*	50 kHz

A required total number of rinduction cells depneds on acclerator parameters. In the example discussed below, it may be two cells from the requirement of energy recovery, assuming an output power of 2.4 kW by a beam of 300 mA.

**Figure 3:**
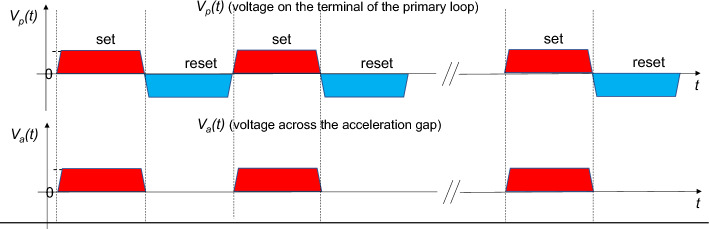
The upper depicts the voltage profiles of set/reset voltage pulses on the primary loop terminal and the lower is that of the accelerating voltage pulses seen by the circulating beam.

Figure 3 shows the set/reset voltage pulse shapes on the primary loop terminal. It is crucial to hide the reset voltage from the circulating beam. The desired pulse shape is realized by the timing of turning on and off the active switching elements. Two shielding switches A and B shown in Fig. 2 are turned on/off in a flip-flop manner. When the set voltage appears on the primary loop terminal, switch A is turned on, closing the corresponding gap, and at the same time, switch B is turned off, resulting in opening of the acceleration gap. Consequently, the accelerating voltage pulses are generated across the insulating gap (acceleration gap) in the beam pipe. When the reset voltage appears on the terminal, switches A and B are turned off and on, respectively, flipping them in the opposite way. The accelerating voltage then disappears. Thus, an accelerating voltage with unipolarity is generated across the gap through the revolution time of the circulating beam.

## Operation of a set of multiple induction cells

**Figure 4:**
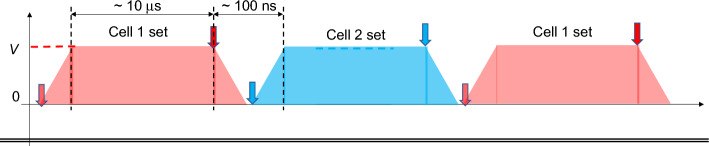
Effective accelerating voltage achieved by operating the set of two induction cells. Arrows represent trigger timing of on/off for the set/reset voltage pulses. Assuming the existing technology^6^, the flat top of 10 ms, rising/falling time of 50 ns, the magnitude of accelerating voltage of 3–4 kV/unit, and repetition rate of a few hundred kHz are realized.

Here, two induction cells are introduced (Cells 1 and 2). If Cells 1 and 2 are continuously operated as a pair in the manner described above with a phase difference π in time, then the circulating beam will be continuously accelerated by the accelerating voltage profile shown in Fig. 4. There is a short time period where the rising set voltage of Cell 2 follows the falling set voltage of Cell 1, and the induction acceleration system cannot provide the necessary accelerating voltage* V*. This is, in a practical sense, an undesirable feature for effective DC acceleration. However, it can be overcome by confining the circulating beam within a finite region by using the barrier voltage pulses employed in existing induction synchrotrons^4,5^. In the example of an isochronous storage ring discussed below, this external confinement is unnecessary. If the repetition rate of the induction acceleration *f*_*in*_ satisfies *f*_*0*_ = *n *× *f*_*in*_ where *n* is integer and *f*_*0*_ is the revolution frequency of the circulating beam, particles in the time region without accelerating voltage will be lost because of frozen phase motions in the isochronous storage ring. As a result, the circulating beam will become a coasting beam with a short gap of missing particles. The gap’s time period of 100 ns has different meanings, depending on the size of circular ring. If its circumference is 30 m, then the transient time period of 100 ns may correspond to simply the time for the circulating beam to make one revolution at the speed of light. This means that the energy compensation is insufficient for the entire beam length to occupy the ring every 100 turns.

It is possible to avoid the accelerating voltage gap every 100 turns by refreshing the circulating beam every 10 ms or 100 turns. Otherwise, a third induction cell could be introduced that is triggered in the transient time period of 100 ns by an induced pulse with a triangular profile consisting of rising and falling so as to fill the lack of voltage. The corresponding reset pulse has no effects because it is triggered away from the transient time region. However, the superimposed voltage profile is expected to deviate slightly from the desired constant accelerating voltage *V*. We will discuss the assessment of the deviation later. By contrast, the 100 ns gap may be rather convenient in the case of much bigger ring, where the third induction cell can be omitted.

## Potential application

The required voltage height and repetition rate for switching will depend on the machine and beam parameters in applications. Practical parameters for the induction cell unit are limited to the following specification: voltage height of < 4 kV/cell due to withstand voltage in the switching device and cell’s inner structure, pulse width of less than a few tens of microseconds due to magnetic core size, and repetition rate of < 1 MHz due to switching loss in the switching device, as stated in the Fig. 4 caption. Note, however, that the required pulse length, voltage height, and repetition rate can be achieved by introducing a multiple induction acceleration system that has been demonstrated previously^6^. For the application introduced here, voltage requirements seem to determine the number of cells required.

A potential application employing this DC acceleration scheme in a circular ring would be a CW terahertz (THz) FEL driven by a coasting beam with an FEL microbunching structure. Observation of the microbunching structure on the RF macrobunch in the storage ring, which is generated as a result of FEL interaction, has been reported previously^9^. A group at Tsinghua University^10^ is planning to construct an isochronous storage ring employing induction acceleration, where a flat long bunch with the FEL microbunch structure will be accelerated by the asymmetric induction acceleration technique^3,11^. In principle, this acceleration technique is similar to that of existing induction synchrotrons, where the accelerating voltage must be generated at the MHz revolution frequency of the beam bunch in most of cases. As the time profile of the amplified wave will inevitably reflect the long beam bunch structure with microstructures, the amplified FEL signal becomes far from a CW.

**Figure 5:**
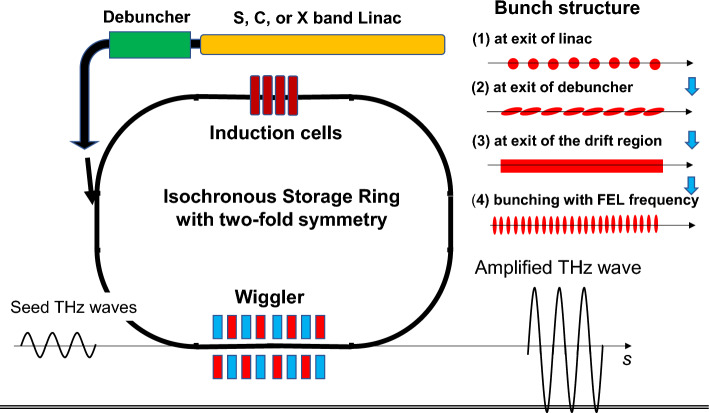
Schematic view of the induction isochronous storage ring CW z FEL. An electron beam, the length of which is a bit shorter than the ring circumference, is accelerated in the linac with the microwave bunching structure. The debuncher operated at the same microwave frequency is placed downstream, at the exit of which the adjacent bunches contact, as seen in the upper wright figure. The flat electron beam is injected into the ring by the injection device placed in the short straight section of the ring. Simultaneously, the THz seed power of kW is introduced from upstream of the wiggler that occupies the long straight section. The flat beam is strongly bunched as a result of FEL interaction. Isochronous of the ring will keep FEL bunching. The FEL bunches continue to take a role to amplify the seed THz waves until refreshing of the electron beam.

Energy loss due to synchrotron radiation and the FEL interaction is compensated by the DC voltage provided in the present induction acceleration scheme. A schematic view is shown in Fig. 5. First, a coasting injection electron beam is generated by the combination of a 14 MeV L, S, C, or X-band linac and a debuncher operated at the same frequency. The beam will be injected into the isochronous storage ring with two-fold symmetry^12^, where two long straight sections are occupied by the wiggler and the induction cells; meanwhile, two short straight sections are used for injection and extraction. THz seed waves are introduced from upstream of the wiggler. FEL micro-bunching of the injected electron beam rapidly progresses, amplifying the injected seed waves. The system will reach a steady state. Thus, the CW THz FEL can be realized. An exhausted beam is extracted from the ring and the driving beam is refreshed. The process is repeated every 100 turns in the example described below. Typical FEL parameters are listed in Table 1, which have been evaluated using the universal gain equation and its analytic solution for a macroparticle representing FEL microbunches, assuming that FEL microbunch is tightly bunched^13,14^. Evaluation of the numerical values in Table 2 is given in the Suppl Appendix. Not that the assumption of a macroparticle is equivalent to that of a zero-emittance beam or bunch. The example shown in Table 2 is the case for an ideal THz FEL.

**Table 2:** Machine/beam/FEL parameters^15^ (nomenclatures follow that in standard accelerator and FEL text books, exceptional ones are defined in this table and Suppl Appendix).

Circumference	*C*_0_	30 m
Energy	*E*	14 MeV
Relativistic *gamma*	*γ*	28.3973
Revolution period	*T*_0_	100 ns
Beam current	*I*_*B*_	300 mA
Betafunction in the wiggler region	*β*_*x*_*/β*_*y*_	3 m
rms beam emittance	*ε*_*x*_*/ε*_*y*_	15 mm-mrad
rms beam size in the wiggler region	*σ*_*x*_*/σ*_*y*_	6.7 mm
Beam cross-section	*S* (*σ*_*x*_ × *σ*_*y*_)	4.5 × 10^–5^ m^2^
Pulse length of induction voltage	*T*	10 μs
THz wave frequency	*f*	*3 THz*
Wavelength	*λ*_*s*_	100 nm
Wavenumber	*k*_*s*_ (*2π/λ*_*s*_)	
Wiggler period	*λ*_*w*_ (2π/*k*_*w*_)	2.7 cm
Saturation distance	*L*_*p*_ = π*/|b|* *a*_*w*_ = *b*_*w*_*/k*_*w*_ *|b|* =*|k*_*w*_* − k*_*s*_*a*^*2*^_*w*_*/2γ*^*2*^*|*	2.23 m
Wiggler length	*L*_*w*_ (= *L*_*p*_)	2.23 m
Required acceleration voltage per turn	$V = \frac{{(Z_{0} \cdot I_{B} )a_{{\text{w}}}^{2} }}{{S \,\, \left| b \right|^{2} }}$	8.235 kV
Amplified power at *z* = *L*_*p*_	*P*_*out*_ = *V* × *I*_*B*_	2.47 kW

Here the above formula for saturation distance is straightforward to evaluate from the analytic solution of the universal gain equation^13^, as shown in the Suppl Appendix.

Here, it is emphasized that this CW FEL is operated not in the SASE mode or oscillator mode, but in amplifier mode. The present scheme is similar to that of the linear multi-stage microwave FEL extensively studied for two-beam accelerators^3,16^, where the driving beam is tightly bunched at the FEL frequency (17.1 GHz) as a result of strong FEL interaction propagating through more than 100 stages, and the amplified microwave with gigawatt output power is extracted by the microwave extraction bend at every stage, and energy loss is replenished in the induction acceleration cell with megavolt voltage placed at the end of a stage. A 1D FEL macroparticle simulation assuming 100 stages has been carried out^17^. At the very least, no fatal degradation was found in the FEL amplification. This finding may encourage feasibility studies of the present scheme for CW THz FELs in the isochronous induction storage ring.

The FEL parameters shown here should be considered just as a reference because the universal gain equation was used with simplified assumptions such as tight bunching, zero transverse emittance, and an intrinsic tapered wiggler (*B*_*w*_/*γ* = const). More precise parameters must be evaluated using modern 3D FEL simulation codes in the context mentioned in the “Discussion”.

## Discussion and summary

It has been long believed that there is no way to continuously accelerate a coasting beam in a circular ring using electrostatic fields. However, it is still possible to realize effective DC acceleration even in such a circular ring by employing an induction acceleration cell with multi-insulating gaps. Key features of the device were described above. Details were explained for operating the induction cell by forced short and open circuits of the insulating gaps. In this paper, solid-state power devices such as SiC-MOFETs were mentioned as an active switching device to allow forced short and open circuits of the insulating gaps. Passive devices such as a SiC diode may be able to serve the same role^18^. This passive device would be much easier to handle from the circuit architecture perspective, since the driving IC circuit for the MOSFET trigger is not needed.

As a typical application of the DC acceleration, a CW THz FEL was proposed, where energy loss of a DC beam occupying the isochronous storage ring including synchrotron radiation is always compensated with an effective DC voltage provided by the proposed scheme. It may be possible to extend the idea of CW THz FEL to a CW extreme ultraviolet (EUV) FEL in a similar but larger scale isochronous induction storage ring by increasing the beam energy to 400–500 MeV. The CW EUV-FEL that generates polarized EUV light and can provide an average power at the kilowatt level would be quite attractive as a next-generation EUV lithography source to replace the present laser ablation EUV source with low wall-plug efficiency of about 0.1%^19,20^. Discussions on details of these CW FEL including the design of isochronous storage ring are beyond the scope of the present study. They will be discussed in forthcoming papers. Important issues that must be carefully investigated to determine the actual feasibility of the proposed scheme are as follows.

1. The required flatness of the DC accelerating voltage
2. The effects of residual deviation in the accelerating voltage
3. The effects of nonlinear magnets for ensuring isochronism of the storage ring
4. Ion trapping by the coasting electron beam
5. Cumulative energy spread as a result of repeated FEL interactions in amplifier mode and degradation in the output power
6. 3D simulations integrating beam motion in the arch region and wiggler region including space-charge effects

These research topics will be big challenges for our accelerator/FEL society to tackle.

To date, the induction acceleration technique has been used in hadron synchrotrons, demonstrating a wide freedom of longitudinal beam handling^6^. It is hoped that induction acceleration using the induction cell with multi-insulating gaps described in this paper will spur new developments in electron synchrotrons and storage rings.

### Supplementary Information

Supplementary Information.

## Data Availability

All data generated and analyzed during the current study are included in the published article.
