# Supplementary material for: Effective DC acceleration of charged particles in a circular ring and its potential application
Source: Sci Rep. 2023 Aug 21;13:13595. doi: 10.1038/s41598-023-40859-2 (PMC10442391; doi:10.1038/s41598-023-40859-2)
Supplement: Supplementary file 1 — Supplementary Information. [file 41598_2023_40859_MOESM1_ESM.docx]

**Appendix**

*The macroparticle approach for the THz FEL* [13]

The magnitude of the amplified THz waves is dominated by the overall behavior of electrons; the THz waves in the FEL strongly couples with the collective motion of the electrons. It is essential to follow the global motion of the bunch. It is assumed that the behavior of the bunch center can represent the collective motion of the bunch. In the employed macroparticle approach (MPA), the macroparticle (MP) located at the bunch center interacts with the seed THz waves to evolve through the wiggler region. FEL equations for the MPA are given in Ref. 13, where the waveguide mode fields are assumed. Since the beam pipe boundary is very far for the THz wave beam, the boundary effects disappear here. In addition, definition of several FEL parameters is different from that in the waveguide mode FELs. The particle equations describe the motion of the MP of energy *γ_a_* in rest-mass units and of the ponderomotive phase *φ_a_=(k_s_+k_w_)z-ω_s_t+ϕ_s_*. The field equations are written in terms of slowly evolving normalized amplitude *e_s_* and the phase shift *ϕ_s_* of the signal wave. The MP FEL equations for THz waves in TE_01_ mode are

*Universal gain equation and its analytic solution* [14]

*Saturation distance L_p_*

*Energy conservation*

*Output power P_out_*

$$\begin{matrix} \text{Energy loss of the macroparticle through the wiggler:} \\ \Delta\gamma=\gamma_{a}\left(0\right)-\gamma_{a}\left(L_{p}\right)=\frac{mc^{2}}{eZ_{0}J\kappa}\cdot\left[e_{s}^{2}\left(L_{p}\right)-e_{s}^{2}\left(0\right)\right]\simeq\frac{mc^{2}}{eZ_{0}J\kappa}\cdot e_{s}^{2}\left(L_{p}\right) \\ \frac{mc^{2}}{e}\cdot\Delta\gamma\cdot I_{B}=\left(\frac{mc^{2}}{e}\right)^{2}\cdot\frac{1}{Z_{0}J\kappa}\cdot\frac{\kappa^{2}}{a^{2}\left|b\right|^{2}}\cdot e^{2y\left(L_{p}\right)}\cdot I_{B} \\ \text{ }=\left(\frac{mc^{2}}{e}\right)^{2}\cdot\frac{\kappa^{2}}{Z_{0}J\kappa}\cdot\frac{1}{4}\cdot\left(\frac{e}{mc^{2}}\right)^{2}\cdot\frac{\left(Z_{0}J\right)^{2}\cdot a_{w}^{2}}{\gamma^{2}}\cdot\frac{e^{2y\left(L_{p}\right)}}{\left|b\right|^{2}}\cdot I_{B} \\ \text{ }=\frac{\kappa\cdot Z_{0}J}{4\gamma^{2}}\cdot\frac{a_{w}^{2}}{\left|b\right|^{2}}\cdot e^{2y\left(L_{p}\right)}\cdot I_{B}=\left[\frac{\kappa\cdot\left(Z_{0}\cdot I_{B}\right)}{2\left(a^{*}b^{*}\right)}\frac{a_{w}^{2}}{\left|b\right|^{2}}\cdot e^{2y\left(L_{p}\right)}\right]\cdot I_{B} \\ \text{Compensated voltage }V\left(=\frac{mc^{2}}{e}\cdot\Delta\gamma\right)\text{ is represented by} \\ V=\frac{\kappa\cdot\left(Z_{0}\cdot I_{B}\right)}{2\left(a^{*}b^{*}\right)}\frac{a_{w}^{2}}{\left|b\right|^{2}}\cdot e^{2y\left(L_{p}\right)}\text{ } \\ \text{where }e^{2y\left(L_{p}\right)}=4,\text{ as proved in the previous page. Assuming }\kappa\simeq\text{1,} \\ \text{Eq. (11) reduces to} \\ V=\frac{\left(Z_{0}\cdot I_{B}\right)}{S}\frac{a_{w}^{2}}{\left|b\right|^{2}}\text{ } \\ \text{where }S=\left(a^{*}b^{*}\right)/2\text{ is the effective area occupied by the THz beam or the electron beam.} \\ \text{Naturally, the output power of the THz FEL is written in the form of} \\ P_{out}=V\cdot I_{B}\text{ (12)} \end{matrix}$$
